# Supplementary material for: Resolving the full spectrum of human genome variation using Linked-Reads
Source: Genome Res. 2019 Apr;29(4):635–45. doi: 10.1101/gr.234443.118 (PMC6442396; doi:10.1101/gr.234443.118)
Supplement: Supplemental Material [file supp_gr.234443.118_Supplemental_longranger-2.2.zip › longranger-2.2.tar.bz2!/lr2.2-public/lib/stan/cmdstan/stan/doc/api/html/dir_f77850045c6d72da1ced64bfc8354271.html]

Stan: src/stan/agrad/rev/functions Directory Reference


|  |  |
| --- | --- |
|  | Stan  2.5.0  probability, sampling & optimization |


- Main Page
- Related Pages
- Namespaces
- Classes
- Files

All Classes Namespaces Files Functions Variables Typedefs Enumerations Enumerator Friends Macros Pages

- src
- stan
- agrad
- rev
- functions

functions Directory Reference

|  |  |
| --- | --- |
| Files | |
| file | abs.hpp [code] |
|  | |
| file | acos.hpp [code] |
|  | |
| file | acosh.hpp [code] |
|  | |
| file | as\_bool.hpp [code] |
|  | |
| file | asin.hpp [code] |
|  | |
| file | asinh.hpp [code] |
|  | |
| file | atan.hpp [code] |
|  | |
| file | atan2.hpp [code] |
|  | |
| file | atanh.hpp [code] |
|  | |
| file | bessel\_first\_kind.hpp [code] |
|  | |
| file | bessel\_second\_kind.hpp [code] |
|  | |
| file | binary\_log\_loss.hpp [code] |
|  | |
| file | cbrt.hpp [code] |
|  | |
| file | ceil.hpp [code] |
|  | |
| file | cos.hpp [code] |
|  | |
| file | cosh.hpp [code] |
|  | |
| file | digamma.hpp [code] |
|  | |
| file | erf.hpp [code] |
|  | |
| file | erfc.hpp [code] |
|  | |
| file | exp.hpp [code] |
|  | |
| file | exp2.hpp [code] |
|  | |
| file | expm1.hpp [code] |
|  | |
| file | fabs.hpp [code] |
|  | |
| file | falling\_factorial.hpp [code] |
|  | |
| file | fdim.hpp [code] |
|  | |
| file | floor.hpp [code] |
|  | |
| file | fma.hpp [code] |
|  | |
| file | fmax.hpp [code] |
|  | |
| file | fmin.hpp [code] |
|  | |
| file | fmod.hpp [code] |
|  | |
| file | gamma\_p.hpp [code] |
|  | |
| file | gamma\_q.hpp [code] |
|  | |
| file | hypot.hpp [code] |
|  | |
| file | ibeta.hpp [code] |
|  | |
| file | if\_else.hpp [code] |
|  | |
| file | inv.hpp [code] |
|  | |
| file | inv\_cloglog.hpp [code] |
|  | |
| file | inv\_logit.hpp [code] |
|  | |
| file | inv\_sqrt.hpp [code] |
|  | |
| file | inv\_square.hpp [code] |
|  | |
| file | is\_inf.hpp [code] |
|  | |
| file | is\_nan.hpp [code] |
|  | |
| file | is\_uninitialized.hpp [code] |
|  | |
| file | lgamma.hpp [code] |
|  | |
| file | lmgamma.hpp [code] |
|  | |
| file | log.hpp [code] |
|  | |
| file | log10.hpp [code] |
|  | |
| file | log1m.hpp [code] |
|  | |
| file | log1m\_exp.hpp [code] |
|  | |
| file | log1p.hpp [code] |
|  | |
| file | log1p\_exp.hpp [code] |
|  | |
| file | log2.hpp [code] |
|  | |
| file | log\_diff\_exp.hpp [code] |
|  | |
| file | log\_falling\_factorial.hpp [code] |
|  | |
| file | log\_rising\_factorial.hpp [code] |
|  | |
| file | log\_sum\_exp.hpp [code] |
|  | |
| file | modified\_bessel\_first\_kind.hpp [code] |
|  | |
| file | modified\_bessel\_second\_kind.hpp [code] |
|  | |
| file | multiply\_log.hpp [code] |
|  | |
| file | owens\_t.hpp [code] |
|  | |
| file | Phi.hpp [code] |
|  | |
| file | Phi\_approx.hpp [code] |
|  | |
| file | pow.hpp [code] |
|  | |
| file | primitive\_value.hpp [code] |
|  | |
| file | rising\_factorial.hpp [code] |
|  | |
| file | round.hpp [code] |
|  | |
| file | sin.hpp [code] |
|  | |
| file | sinh.hpp [code] |
|  | |
| file | sqrt.hpp [code] |
|  | |
| file | square.hpp [code] |
|  | |
| file | step.hpp [code] |
|  | |
| file | tan.hpp [code] |
|  | |
| file | tanh.hpp [code] |
|  | |
| file | tgamma.hpp [code] |
|  | |
| file | trunc.hpp [code] |
|  | |
| file | value\_of.hpp [code] |
|  | |


---

[ Stan Home Page ]

*© 2011–2014,
Stan Development Team.*
